# Biophysical and biochemical constraints imposed by salt stress: learning from halophytes

**DOI:** 10.3389/fpls.2014.00746

**Published:** 2014-12-22

**Authors:** Bernardo Duarte, Noomene Sleimi, Isabel Caçador

**Affiliations:** ^1^Centre of Oceanography, Faculty of Sciences, University of LisbonLisbon, Portugal; ^2^Marine and Environmental Sciences Centre, Faculty of Sciences, University of LisbonLisbon, Portugal; ^3^UR: MaNE, Faculté des Sciences de Bizerte, Université de CarthageBizerte, Tunisie

**Keywords:** photochemistry, halophytes, oxidative stress, osmoregulation, stress, physiological

## Abstract

Soil salinization is one of the most important factors impacting plant productivity. About 3.6 billion of the world’s 5.2 billion ha of agricultural dry land, have already suffered erosion, degradation, and salinization. Halophytes are typically considered as plants able to complete their life cycle in environments where the salt concentration is above 200 mM NaCl. Salinity adjustment is a complex phenomenon but essential mechanism to overcome salt stress, with both biophysical and biochemical implications. At this level, halophytes evolved in several directions, adopting different strategies. Otherwise, the lack of adaptation to a salt environment would negatively affect their electron transduction pathways and the entire energetic metabolism, the foundation of every plant photosynthesis and biomass production. The maintenance of ionic homeostasis is in the basis of all cellular counteractive measures, in particular in terms of redox potential and energy transduction. In the present work the biophysical mechanisms underlying energy capture and transduction in halophytes are discussed alongside with their relation with biochemical counteractive mechanisms, integrating data from photosynthetic light harvesting complexes, electron transport chains to the quinone pools, carbon fixation, and energy dissipation metabolism.

## INTRODUCTION

If we take a good look to our planet we will conclude that it is in fact a salt planet. About 70% of its surface is covered by salt water, with concentrations of Na^+^ around 500 mM and contrasting low K^+^ concentrations of 9 mM (Flowers, 2004). Alongside, the remaining 30% of the Earth’s surface is severely affected by increased salinization, enhanced by improper agricultural soil use and irrigation practices (Zhang and Shi, 2014). We live in a time of changes, the ongoing climate-driven changes must also be considered as well as their consequences, such as increasing drought frequency and intensity, air temperature, and salt water intrusion in coastal soils (Duarte et al., 2013a). All these aspects impose severe constraints to the primary production of Earth, namely crop production. Salinity-induced constraints in plants are associated with reductions in leaf expansion, stomatal closure, reduced primary production, biomass losses, and nutritional deficiencies, like K^+^ deficiency (Mahajan and Tuteja, 2005; Rahnama et al., 2010; James et al., 2011). Halophytes are an exception, being highly productive under saline conditions.

The typical definition of halophyte is a plant species that can survive and reproduce under growth conditions with more than 200 mM NaCl (Flowers and Colmer, 2008). Some of these species can be classified as ‘obligatory halophytes’ like *Suaeda maritima* and *Mesembryanthemum crystallinum* requiring saline environments for optimal growth, while other species like *Puccinellia maritima* and *Thellungiella halophila* are included in the group of the so-called “facultative halophytes” with optimal growth without salt in the substrate though tolerating high NaCl concentrations (Flowers, 1972; Gong et al., 2005; Gao et al., 2006; Agarie et al., 2007; Wang et al., 2007, 2009; Guo et al., 2012). The survival and productivity of these species outcomes from a complex network of mechanisms involving multiple biochemical and physiological traits of salt tolerance. Over the last decades, this issue attracted several research groups since the global soil salinization problem became more and more widespread, increasing the need to understand these mechanisms with the main objective of transposing this knowledge to economically relevant crops. Simultaneously, some halophytes were identified as potential nutritional sources with high nutritional value and possibilities to be cultivated in arid environments of the poorer regions of the planet. Several halophytes were already identified and used commercially as food sources like *Aster tripolium* (Ventura et al., 2013), *Chenopodium quinoa* (Eisa et al., 2012), and *Salicornia sp*. (Ventura and Sagi, 2013).

Several reviews have been published focusing at salt stress in halophytes and glycophytes from all over the world and in several different ecosystems. More than describing the anatomical and biochemical adaptations of different halophytes and halophytic strategies to salinity, the present work intends to connect these traits with the most recent biophysical approaches, relating adaptions, and stress signs with the cellular redox homeostasis and bioenergetics. The features are at the basis of the primary production, so knowledge of these bioenergetics traits can provide powerful insights for understanding salt stress in glycophytic crops as well as new opportunities for the improvement of their salinity tolerance.

## ANATOMICAL MODIFICATIONS

**FIGURE 1:**
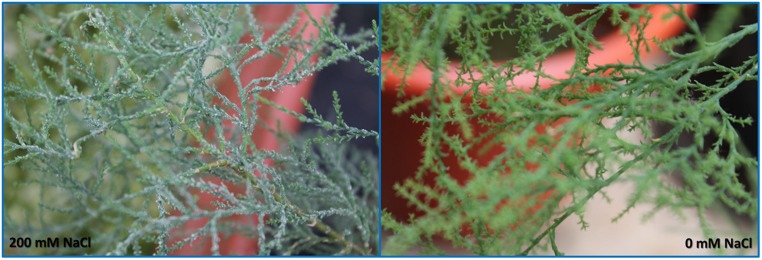
***Tamarix gallica* leaves of individuals subjected to 200 and 0 mM NaCl Photo by B. Duarte (2012).** Plants were originally collected in Tunisia and transplanted to the Centre of Oceanography greenhouse, where they were subjected to different salinity levels.

**FIGURE 2:**
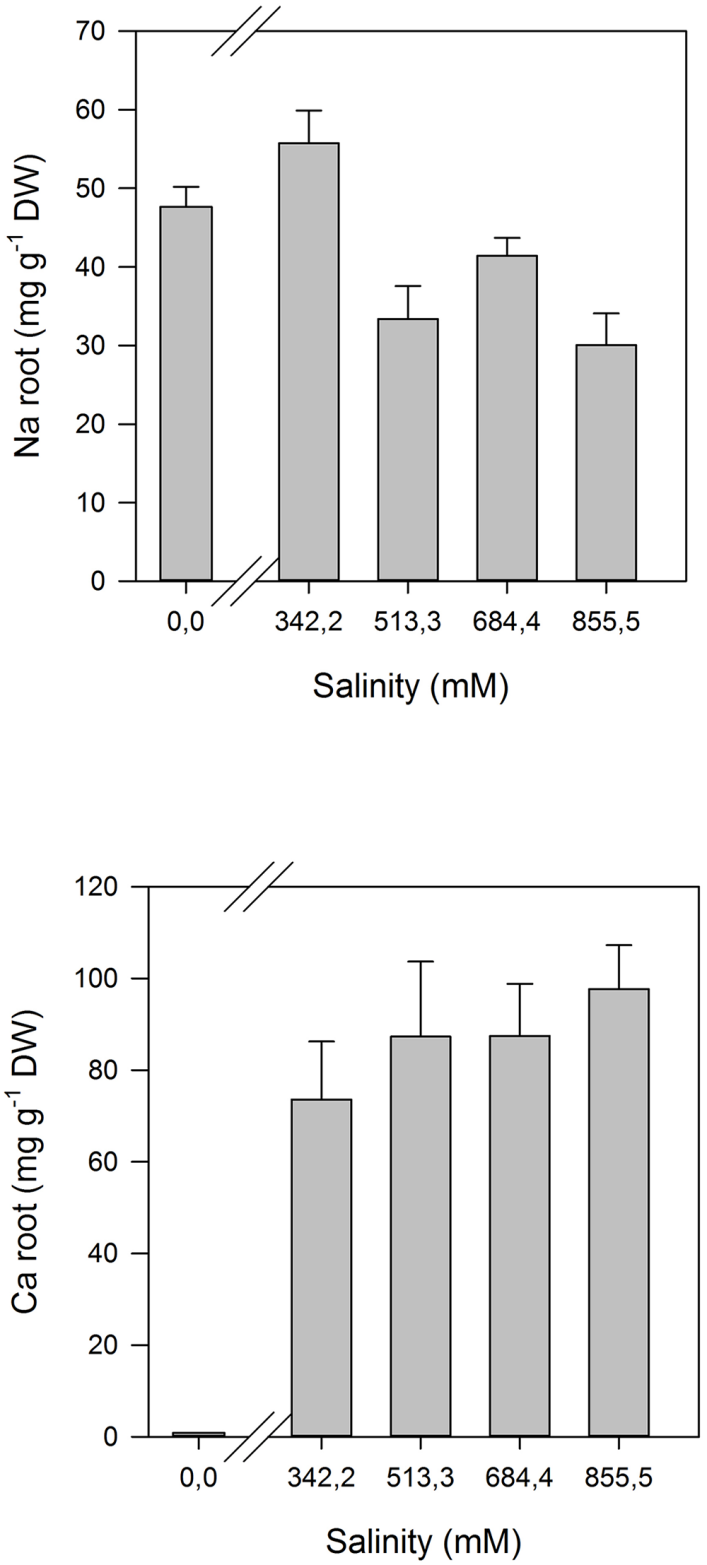
**Na^+^ and Ca^2+^ ionome in the roots of *Sarcocornia fruticosa* exposed to increased salinity levels (average ± SE, *N* = 5)**.

**FIGURE 3:**
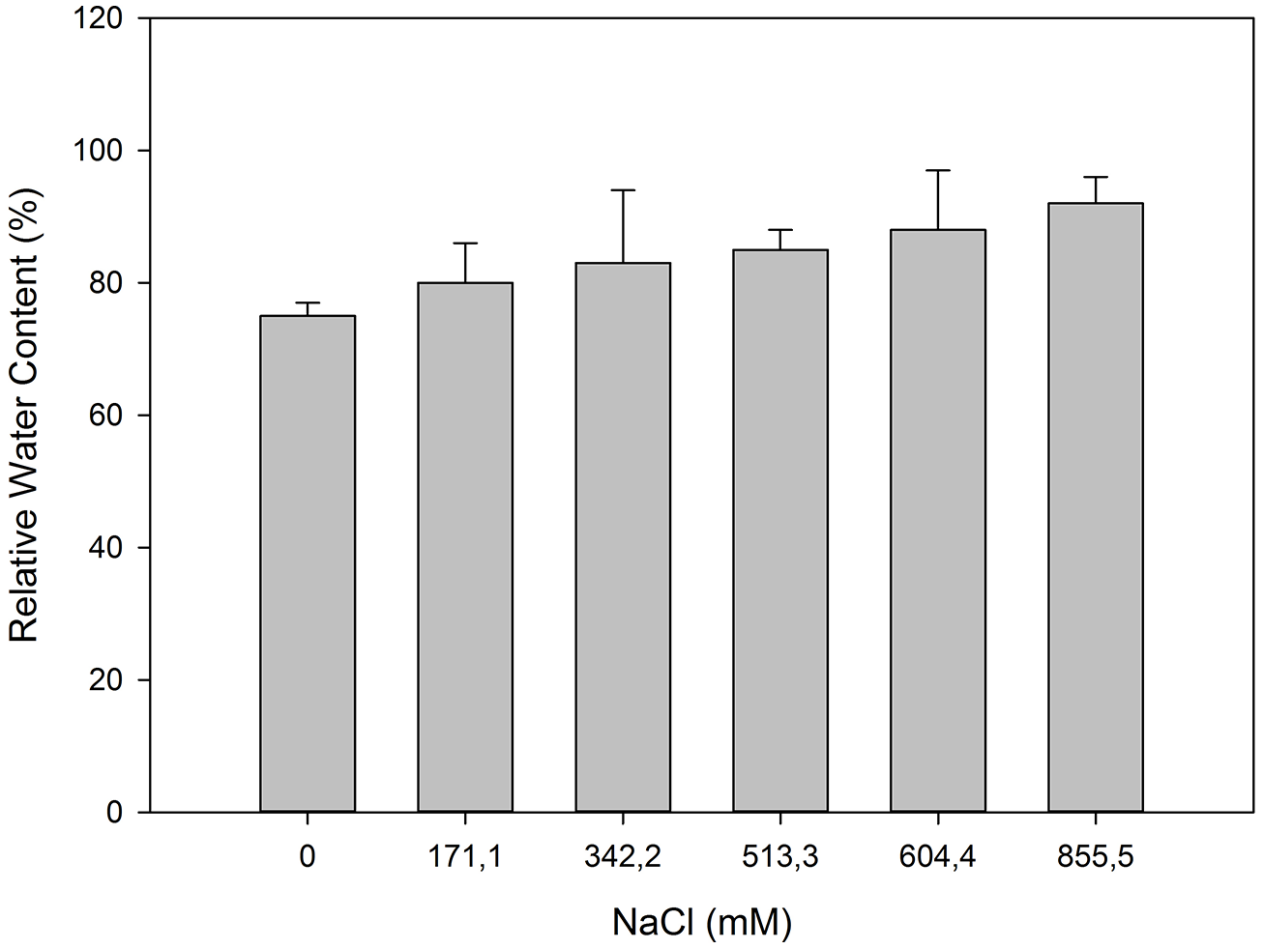
**Relative water content in photosynthetic stems of *S. fruticosa*, which was exposed to increased salinity levels (average ± SE, *N* = 5)**.

Some of the evident adaptations to salt environments can be immediately detected just observing halophyte morphology. Typically, there are two mechanisms that halophytes use in order to overcome high salinity: secretion and exclusion. The secretion-based strategy implies the existence of specialized salt glands (**Figure 1**), located at the leaf surface. The main function of salt glands is the excretion of excessive Na^+^ (Shabala et al., 2014) as a way to reduce its negative effects on cell metabolism. This is probably the most studied tolerance adaptation mechanism in halophytes (Rozema et al., 1981; Waisel et al., 1986; Shabala et al., 2014). The excreted salt crystals on the leaf surface are then washed out by rain or tidal waters, preventing its reabsorption to the leaf cells (Balsamo et al., 1995). On the contrary a typical halophyte excluder retains high amounts of K^+^ and Ca^2+^ inside its cells to avoid Na^+^ uptake, enabling survival in soils with very high salt concentrations (**Figure 2**). The increased Ca^2+^ concentrations allow the cell membrane to maintain the K^+^/Na^+^ selectivity and thus maintain the ionic balance of the cell (Cramer et al., 1987). Alongside with this shoot-exclusion, there is often an observable increase in root Ca^2+^ concentration accompanied by a decrease of the Na^+^ root concentration. This exclusion strategy is well studied in *Sarcocornia fruticosa,* frequently followed by a dilution strategy, implying an increased cellular water uptake and thus decreasing the ionic concentration inside the cell (**Figure 3**). *T. halophila* also evidences a very similar strategy, retaining higher K^+^ and lower Na^+^ concentrations, while increasing its water uptake (Volkov and Amtmann, 2006). This differential ionic absorption is mediated by specific protein ionic channels, with a total of 32 salt induced differentially expressed proteins already identified in *T. halophila* (Pang et al., 2010). Under stress, K^+^ transporter proteins are preferentially expressed alongside with changes in membrane potential and ion selectivity, counteracting the elevated extracellular Na^+^ concentrations. Nevertheless, all these morphological adaptations have implications at both biophysical and biochemical levels.

## BIOPHYSICAL FEEDBACK

**FIGURE 4:**
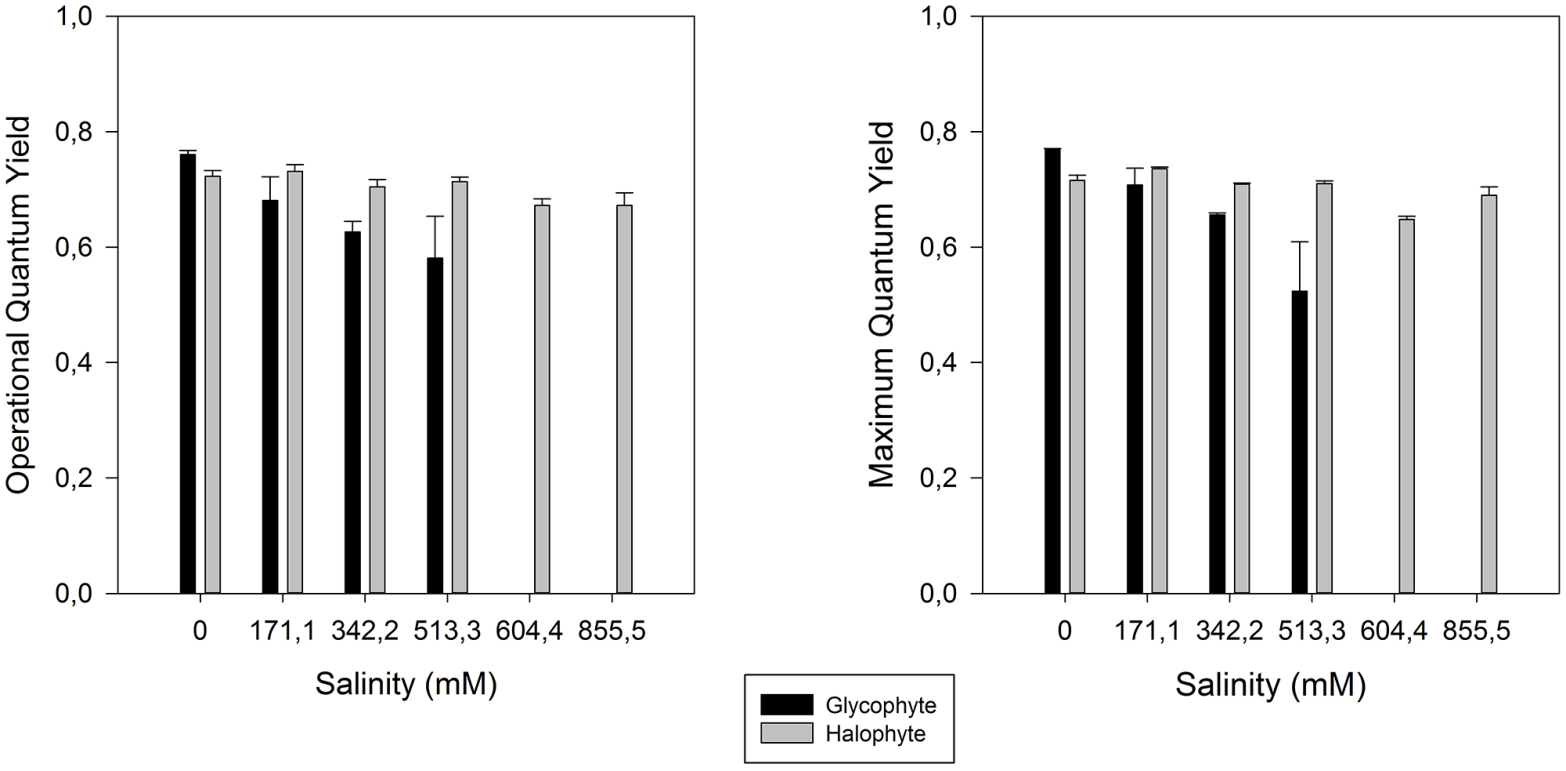
**Operational and maximum PSII efficiency in glycophyte *Cyperus longus* and in halophyte *Spartina patens* along a salinity gradient (average ± SE, *N* = 5)**.

**FIGURE 5:**
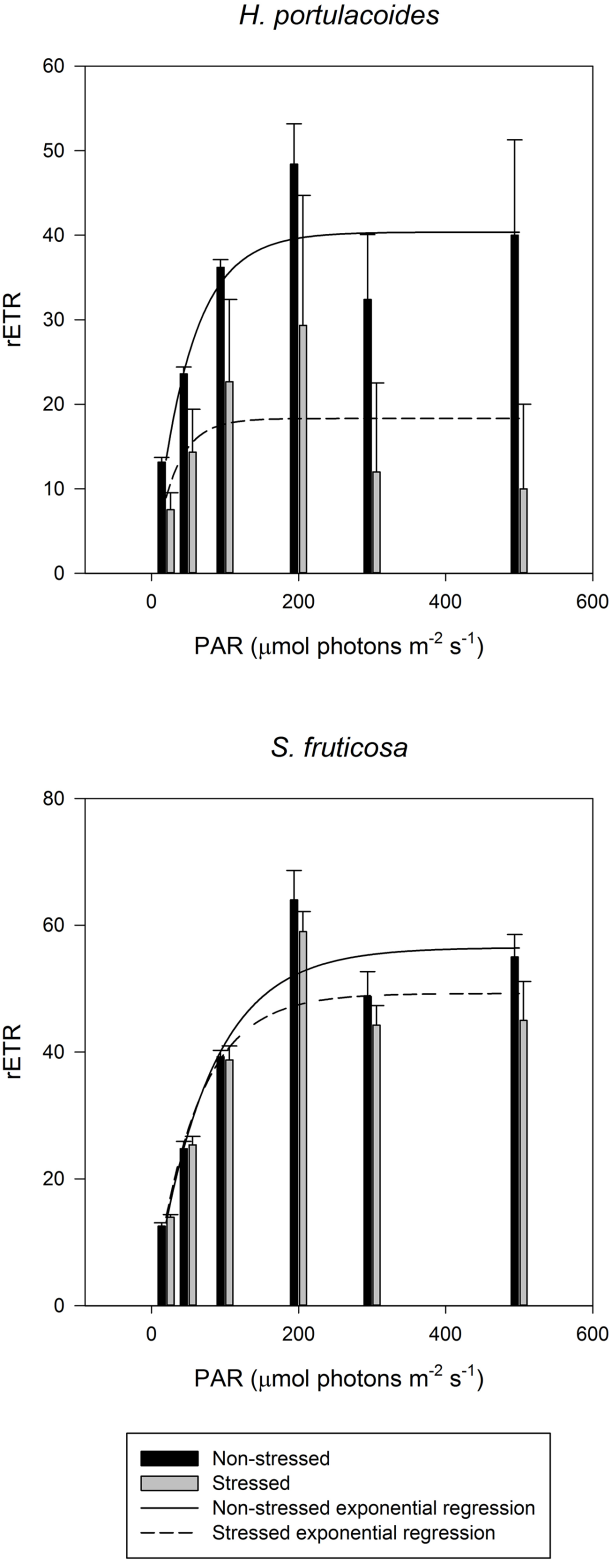
**Electron transport rate (rETR) at different light intensities in field stressed (gray) and non-stress individuals (black) of *Halimione portulacoides* and *S. fruticosa* (average ± SE, *N* = 5)**.

As all other excessive ionic accumulation, excessive salinity has also redox implications at the cellular level, unbalancing the cellular electron fluxes. A decrease in the photosynthetic capacity is very common in salt stressed plants (Munns and Termaat, 1986; Munns, 1993; Qiu et al., 2003; Jaleel et al., 2007), mostly due to a low osmotic potential of the soil solution (osmotic stress), specific ion effects (salt stress), nutritional imbalances, or more usually, a combination of all these factors (Zhu, 2003). One of the consequences of salinity-induced photosynthetic impairment is the exposure of plants to excess of light energy and its inevitable consequences for the photosystem II (PSII). Plants under salt stress use less light energy for photosynthesis (Megdiche et al., 2008). Therefore the presence of efficient energy dissipation mechanisms is essential in order to prevent the accumulation of excessive energy within the cells in the form of excessive reducing potential (Demmig-Adams and Adams, 1992; Qiu et al., 2003). Salinity constraints for photosynthesis are not restricted to the light harvesting processes. Also the photosynthetic carbon fixation reactions are affected under salt stress, mostly due to disturbances of leaf osmotic potential, of the chloroplast membrane systems and of pigment composition (Munns, 2002; Zhao et al., 2007). To avoid damage in the PSII, plants have developed several strategies to dissipate excessive energy. Comparing the PSII activity of glycophytes (*Cyperus longus* for example) with halophytes (*Spartina versicolor* for example) in a salt medium, the differences are evident (**Figure 4**). In glycophyte species, both real (operational) and maximum PSII activities suffer drastic decreases under salt stress. On the other hand, halophytic species, well adapted to salt environments, show almost no differences along a salinity gradient even under oceanic salt concentrations. PSII quantum yield provides rapid and valuable insights on the overall PSII energetic processes. Nevertheless, in order to understand the causes behind these changes, as well as the mechanisms that allow halophytes to overcome salt stress, we need to take a closer look into the biophysics and energetics of the chloroplast. PSII efficiency relies essentially on two major processes: (1) photon harvesting, entrapment and energy transfer throughout the transport chain and (2) dissipation of excessive reducing power. The delicate balance between both these processes is important for all the electron transduction pathway and evidently for energy production. Overlooking the first one, and focusing especially in the electron transport processes, two strategies can be observed depending on the plant tolerance and mechanisms of the salt tolerance (**Figure 5**). Observing the rapid light curves obtained for *Halimione portulacoides* (excretion strategy) and *S. fruticosa* (exclusion strategy), the differences are evident. Although the exclusion strategy of *S. fruticosa* takes place in the roots, this will condition the Na^+^ translocation for the aboveground organs. Nevertheless excessive Na^+^ translocation can still happen and in this case the swelled photosynthetic steams will act as sinks, storing Na^+^ in their vacuoles (Flowers and Colmer, 2008). In *S. fruticosa* the maximum electron transport rate (ETR_max_), photosynthetic efficiency and the onset of light saturation are very similar between control and stressed individuals, with only small differences in the ETR at some light levels. On the other hand, *H. portulacoides* stressed and control individuals exhibited very distinct photosynthetic parameters. Not only the photosynthetic efficiency and the onset of light saturation were reduced to nearly zero, but also the ETR_max_ was severely decreased in stressed individuals. Observing *S. fruticosa* control and stressed individuals we found no major differences neither between the ETR nor in the onset of light saturation, indicating a normal functioning in the ETC. As for *H. portulacoides*, not only the ETR was rather decreased in stressed individuals, but these individuals also have a smaller onset for light saturation, indicating an incapacity to use the absorbed photons for primary photochemical purposes. This inevitably leads to an accumulation of large amounts of reducing power with a high potential for reactive oxygen species (ROS) generation that, as stated before, can destroy the D1 protein, impairing the photochemical apparatus (Rintamäki et al., 1995). Again, two tolerance mechanisms are evidenced between these two *Amaranthaceae* species. *S. fruticosa* presents a salinity tolerance mechanism that allows the PSs to absorb light even under high Na^+^ concentrations. On the other hand, in *H. portulacoides* these mechanisms appear to be absent or inactivated, leading to lower light harvesting and carbon fixation efficiencies. In fact *S. fruticosa* exhibits a common feature among halophytes with an improvement of some energy conversion mechanisms under elevated salt concentrations (Mateos-Naranjo et al., 2010; Rabhi et al., 2012). Diving even deeper in the electron transfer processes, it is possible to understand how the energy fluxes, which result in the total overall PSII activity, are affected by salt stress.

**FIGURE 6:**
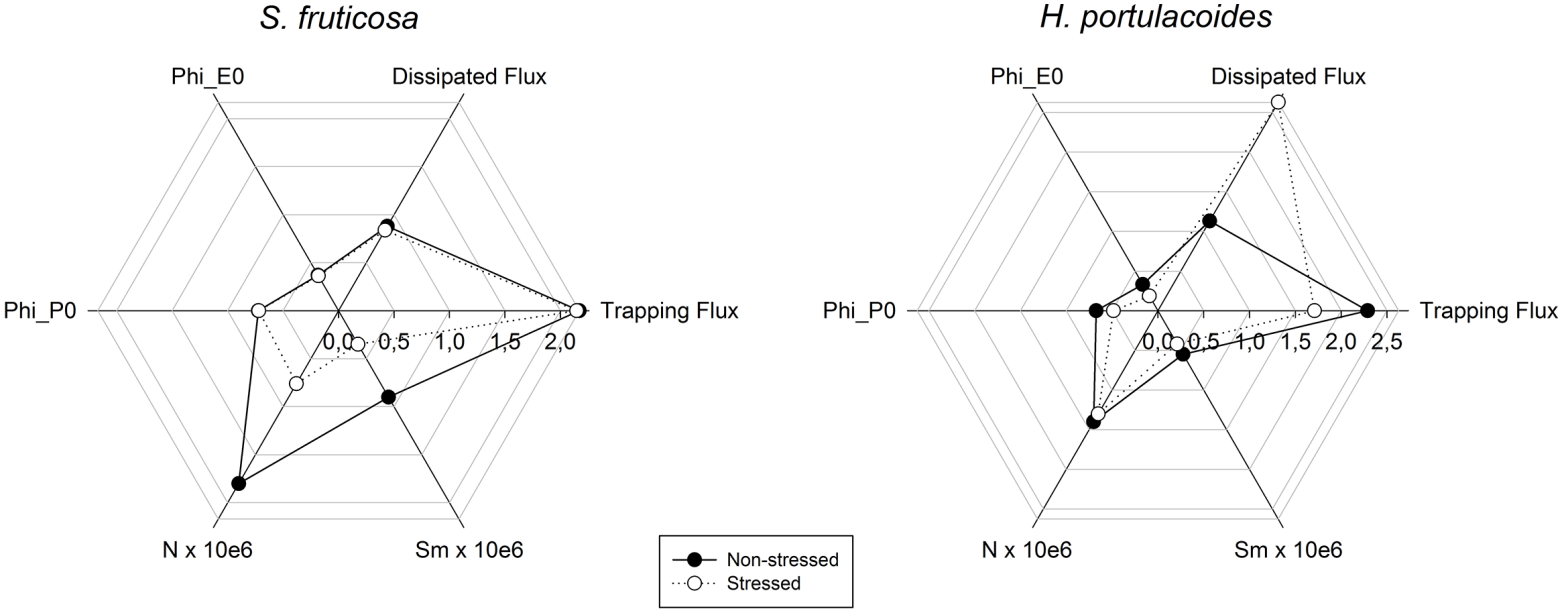
**Rapid transient OJIP curve calculated parameters (Phi P_0_, maximum yield of primary photochemistry; Phi E_0_, probability that an absorbed photon will move an electron into the ETC; N, the number of quinone turnovers until maximum fluorescence is attained; Sm, the number of electrons that flow from the quinones to the ETC) radar plot in field stressed (white) and non-stressed individuals (black) of *S. fruticosa* and *H. portulacoides* species (average values, *N* = 5)**.

A closer investigation of the photochemical mechanisms (**Figure 6**) shows that in *S. fruticosa* the salinity adverse effects are mostly felt at the quinone level, affecting both the electron flow from reduced quinone to the electron transport chain (ETC) and also the quinone pool (Sm). Sm and the quinone reduction turnover rate (N) were severely reduced (**Figure 6**), leading to an excessive accumulation of reduced compounds and low redox potential (Kalaji et al., 2011). In *H. portulacoides*, the negative effects driven by salt stress result in lower light use efficiencies (LUE) due to high amounts of dissipated energy (Rintamäki et al., 1995). In these individuals, alongside with a lower probability that an incident photon can initiate an electron transfer via the ETC there is also a reduced efficiency for a trapped electron to move further than the oxidized quinone. This leads to an inevitable reduction in the maximum yield of primary photochemical processes (Kalaji et al., 2011).

**FIGURE 7:**
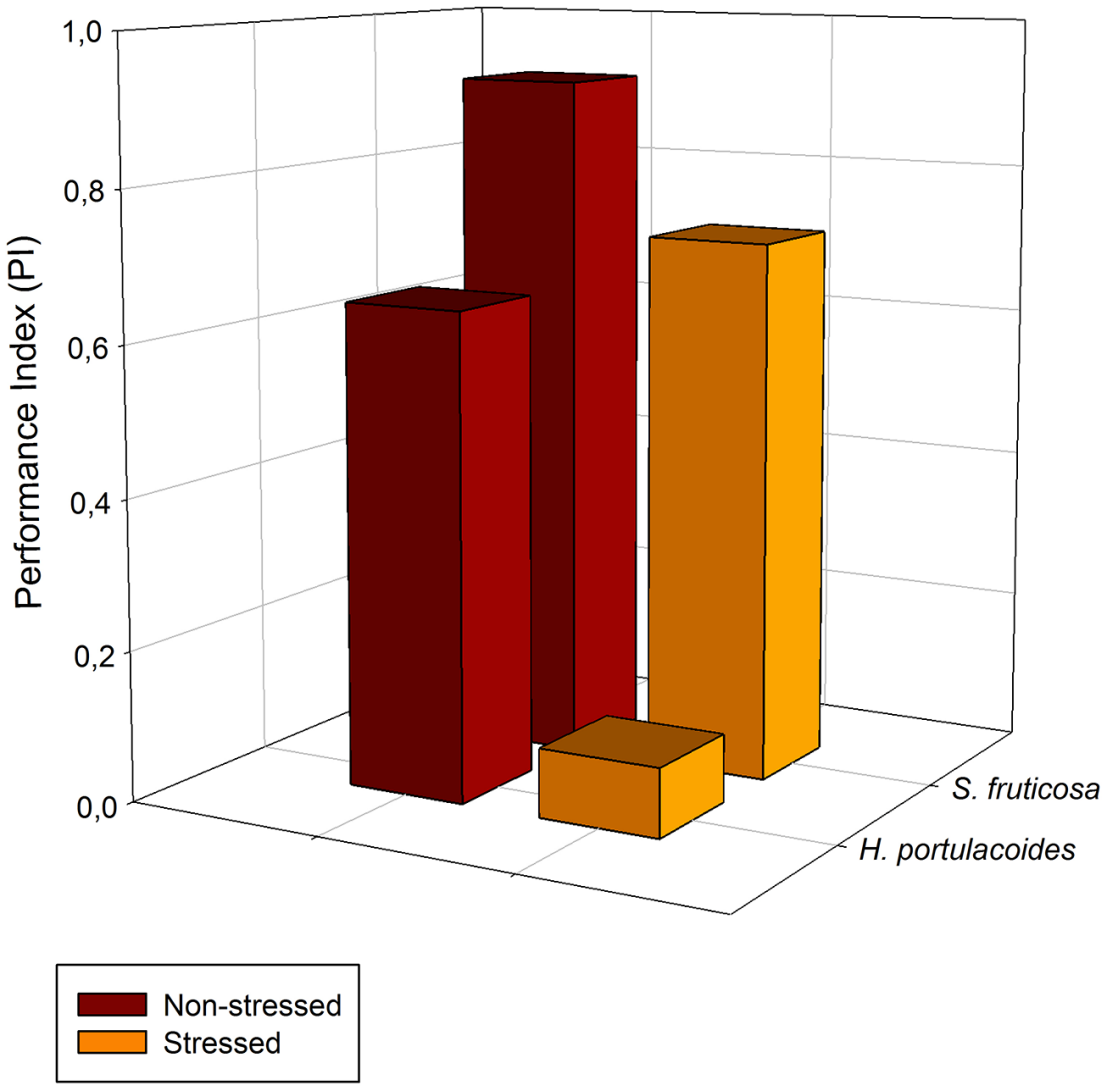
**Performance Index (PI) derived from the OJIP-test, in field stressed (orange) and non-stressed individuals (brown) of *S. fruticosa* and *H. portulacoides* species (average values, *N* = 5)**.

**FIGURE 8:**
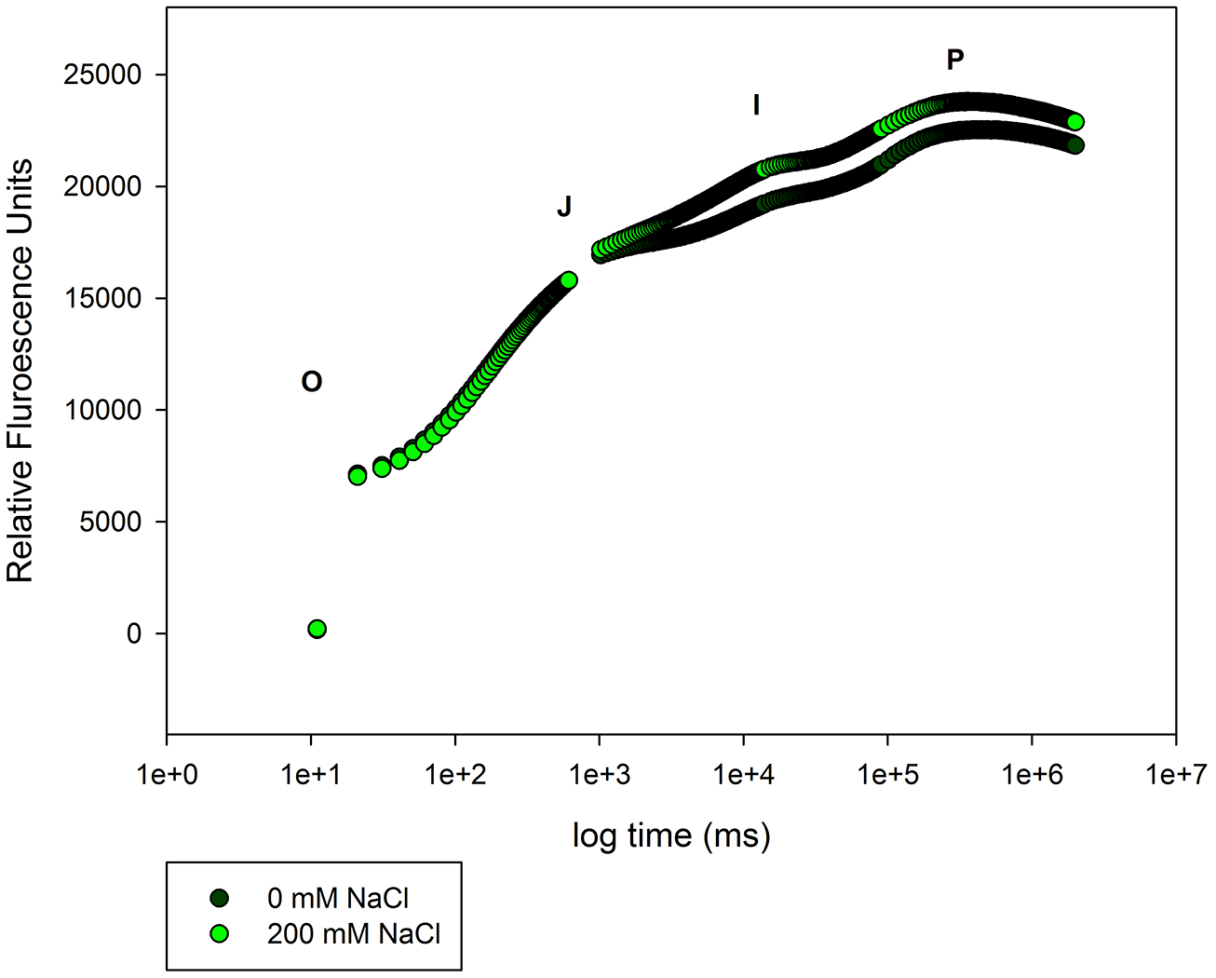
**Kautsky curve from *T. gallica* individuals exposed to 0 and 200 mM NaCl, during 20 days (average values, *N* = 5)**.

**FIGURE 9:**
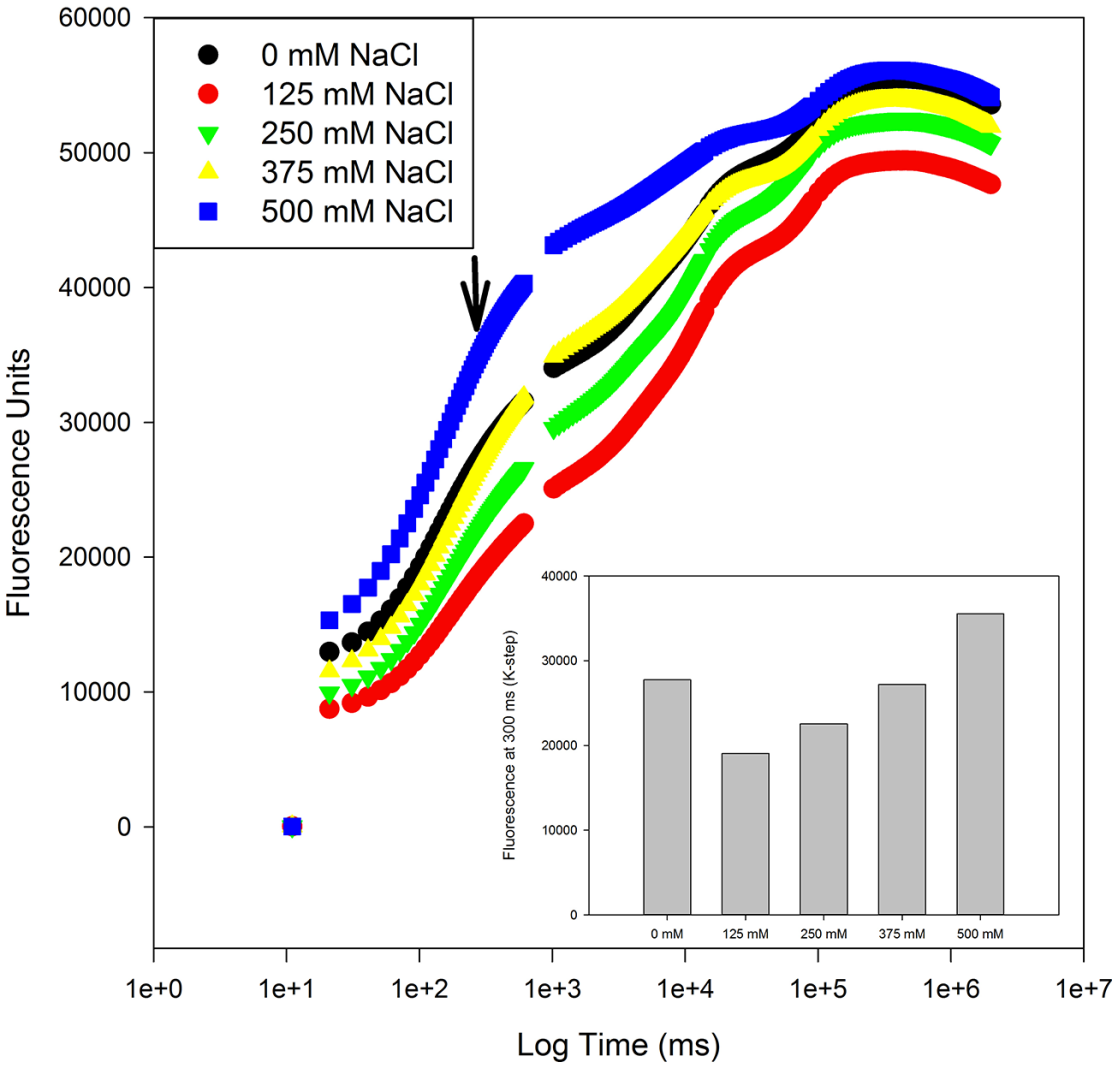
**Kautsky curve and K-step fluorescence (arrow) from *Aster tripolium* individuals exposed to a salinity gradient during 15 days (average values, *N* = 5)**.

A special group of fluorescence parameters derived from high-resolution measurements analysis of the chlorophyll *a* fluorescence kinetics, can offer detailed information on the structure and function of plant photosynthetic apparatus, mainly PSII. Analysis of O-J-I-P fluorescence transient by the JIP-test (Strasser et al., 1995) can be applied to derive a number of parameters quantifying the flow of energy through the PS II both at the reaction centre (RC) and at excited cross-section (CS) levels. This approach is far more sensitive that the traditional PSII quantum yields, being able to detect stress symptoms even before they are visible (Force et al., 2003; Christen et al., 2007). Strasser et al. (2000) also created a Performance Index (PI) to sum all the major processes within the JIP-test in order to express the plant vitality. This integrative parameters includes three independent variables: density of fully active RCs, efficiency of electron transfer generated by an exciton into the ETC and beyond the oxidized quinone pool (Q_A_), and the probability that an absorbed quanta is trapped within the RCs. This way, PI reflects the functionality of both PSI and II and produces quantitative information of the plant performance, especially under stress conditions (Strasser et al., 2004). In the present case, although excessive salt produces negative effects at different levels in both species, all these effects can be well summarized in the reduced PI observed in stressed individuals (**Figure 7**). This PI reduction outcomes from its dependence on the primary photochemical and energetic yields. The behavior exhibited by *S. fruticosa* can be easily measured using a rapid induction Kautsky curve and is very similar to the one found in *Tamarix gallica* when supplied with 200 mM NaCl (**Figure 8**). This type of analysis is very quick and allows a rapid interpretation of the overall energetic fluxes underlying the PSII activity. In this assessment two phases can be distinguished: O-J step or photochemical phase and the J-I-P step or thermal phase. The first one is considered to be a good proxy of the photochemical energy production realized inside the chloroplasts, while the second one reflects the ability to dissipate excessive amounts of energy throughout thermal dissipation. It is possible to observe that *T. gallica* individuals have similar photochemical activity both with and without salt, but the individuals supplemented with 200 mM NaCl have a higher ability to dissipate excessive energy. This is one of the most common mechanisms by which halophytes overcome the accumulation of excessive reducing power, the primary source of ROS, avoiding this way the photo-destruction of the photosynthetic apparatus (Duarte et al., 2013b). Another interesting phenomenon observable while analyzing the Kautsky curves, is the appearance of a new phase, called K-step at 300 μs (**Figure 9**). The appearance of this K-step with salt stress is associated with damage in the PSII donor side mostly at the level of the oxygen-evolving complexes (Srivastava et al., 1997; Strasser and Stirbet, 2001; Strasser et al., 2004; Chen and Cheng, 2009). This is evident in *A. tripolium* exposed to different salt concentration and is normally indicative of a low stability of the oxygen evolving complexes (OECs) under excessive salt concentration, similarly to what was previously observed in plants subjected to thermal stresses (Wen et al., 2005).

## BIOCHEMICAL RESPONSES

**FIGURE 10:**
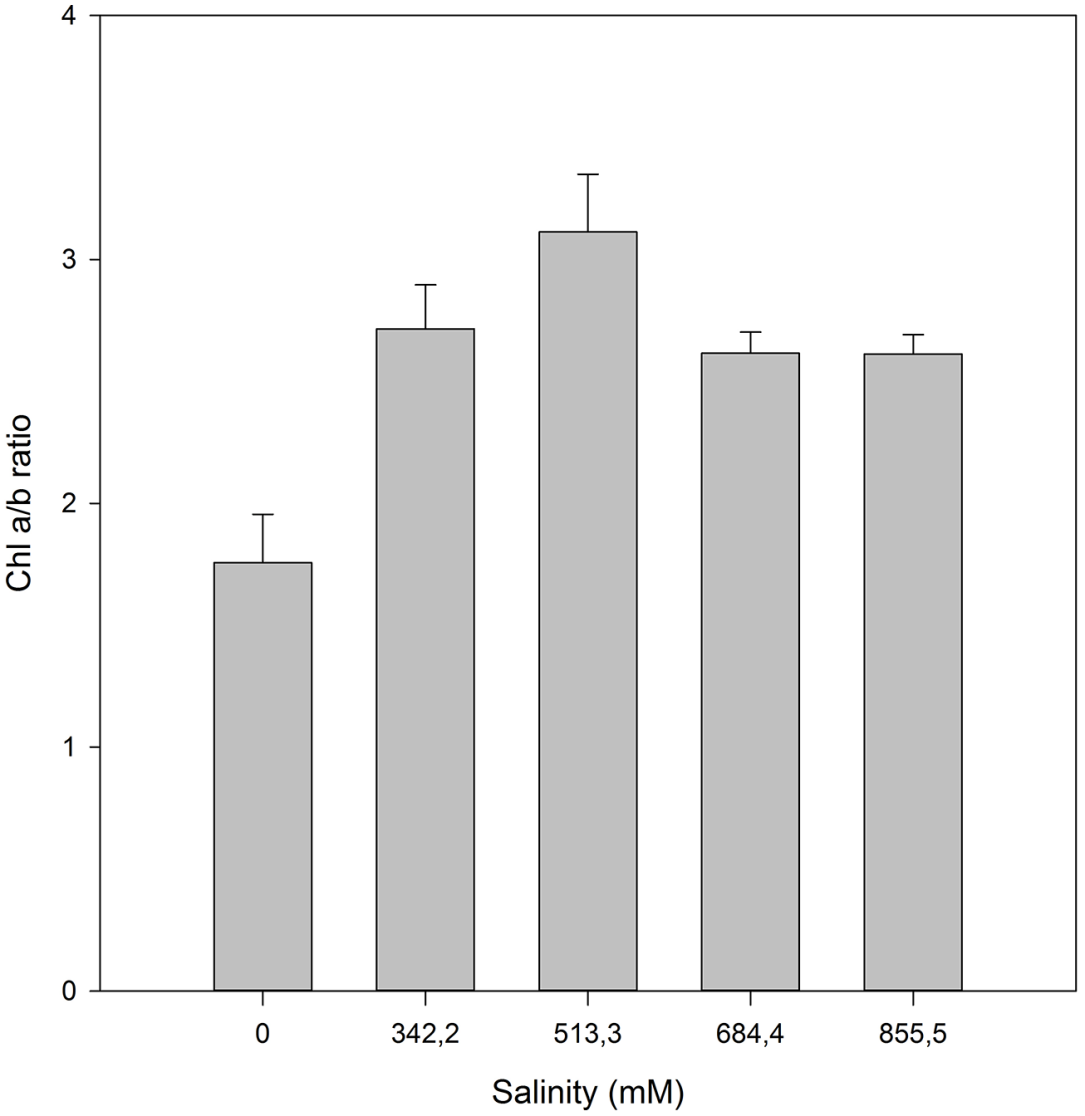
**Chlorophyll a/b ratio in *H. portulacoides* leaves from individuals exposed to a salinity gradient (average ± SE, *N* = 5)**.

Beyond the biophysical processes, halophytes also have a battery of biochemical adjustments to counteract, at the molecular level, the cellular stress imposed by excessive ionic concentrations, namely Na^+^. Still discussing the photosynthetic light harvesting mechanisms: the pigment profiles are frequently affected by elevated salt concentrations. On the other hand, under favorable conditions, the increased PS efficiency, driven by optimal salt concentrations is accompanied by a decrease of the PSII antenna size. Due to the lower requirements for light harvesting at optimum conditions, there is a reduction in the plant needs for larger light harvesting complexes (LHC) oppositely to the observed under stress conditions (Rabhi et al., 2012). This can be evaluated using the chlorophyll a/b ratio as proxy (**Figure 10**). An increase in the chlorophyll a/b ratio is directly related to higher number of active light harvesting RCs, being commonly used as indicator of an enhancement in the plant photochemical capacity. On the other hand, when the halophyte is out of its saline comfort concentrations, the excessive energy reaching the photo-systems must be dissipated (Duarte et al., 2013b). *H. portulacoides* appears to have a physiological optimum at median NaCl concentrations (513.3 mM) similar to those observed in its natural habitat (estuarine salt marshes).

**FIGURE 11:**
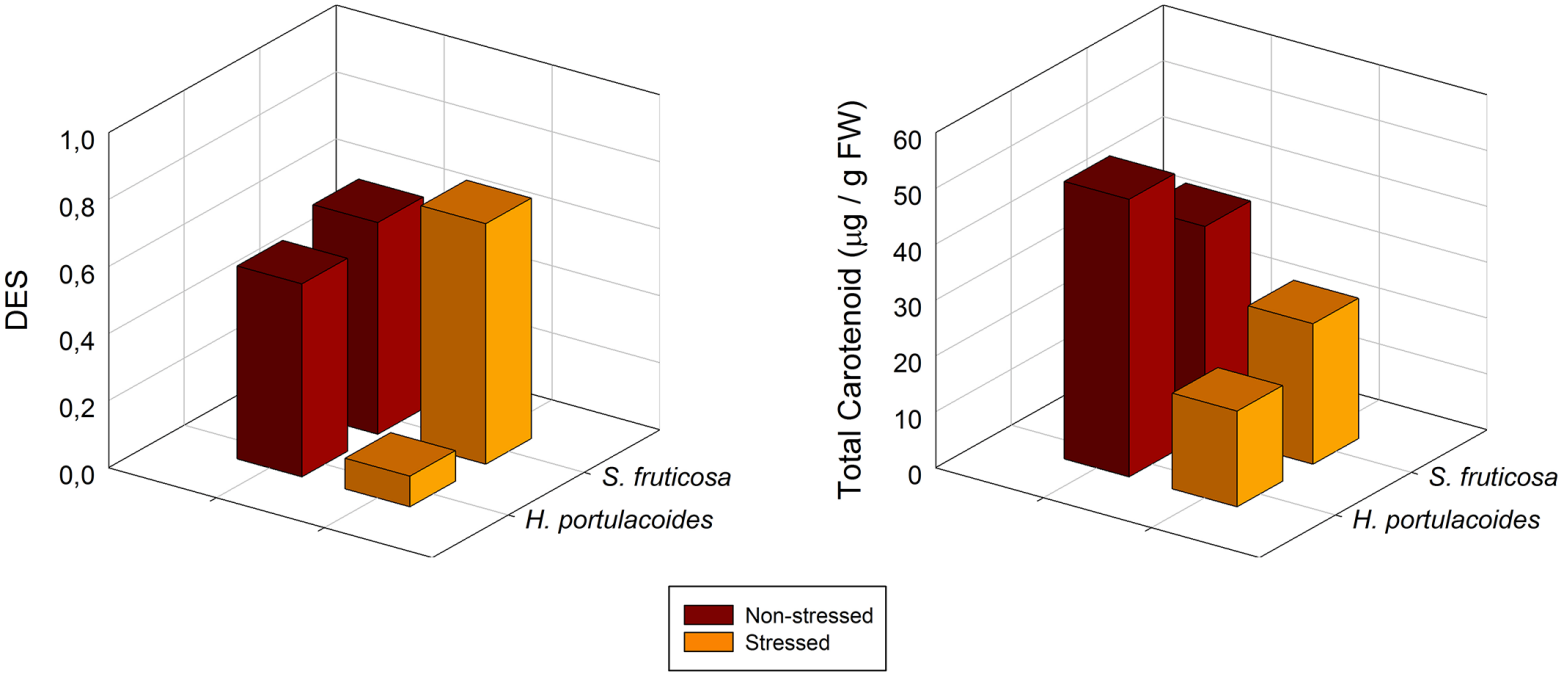
**De-epoxidation state (DES) and total carotenoids in *S. fruticosa* and *H. portulacoides* field stressed (orange) and non-stressed individuals (brown; average ± SE, *N* = 5)**.

Nevertheless, this increase in LHC is sometimes not sufficient to sustain all the incoming solar radiation. At this moment, the plant needs to dissipate the energy in excess, either by fluorescence quenching or throughout a pigment metabolic pathway involving a class of carotenoids called xanthophylls (Demmig-Adams and Adams, 1992). As abovementioned, the salt stressed plants cannot withstand a usual dose of light as in a normal situation, and thus even at low solar radiances it undergoes photo-inhibition increasing the energy dissipation needs. An evident signal of environmental stress is enhanced activation of the xanthophyll cycle, revealed by an increase in the De-Epoxidation State (DES) index (**Figure 11**). When the absorbed light exceeds the plant photochemical capacity (as revealed above by the decrease in the chl a/b ratio), this excessive energy may be transferred to the ever-present oxygen, generating ROS. These molecules affect many cellular functions by damaging nucleic acids, oxidizing proteins, and causing lipid peroxidation (Gill and Tuteja, 2010). Under steady state conditions, the ROS molecules are scavenged by various antioxidative enzymatic and non-enzymatic defense mechanisms (Foyer and Noctor, 2005). In this context, the conversion of violaxanthin to zeaxanthin throughout the xanthophyll cycle is considered to be one of the most effective energy dissipation mechanisms (Demmig-Adams and Adams, 1992). Zeaxanthin may be an important antioxidant in the thylakoid membrane bilayer itself, where it could scavenge ROS and/or terminate lipid peroxidation chain reactions (Muller et al., 2001).

Also the total chlorophyll to total carotenoids ratio, points out in the same direction. In stressed individuals it is common to observe an increase in this ratio, indicating lower chlorophyll concentration, enhancing photo-protection in detriment of light harvest (**Figure 11**).

**FIGURE 12:**
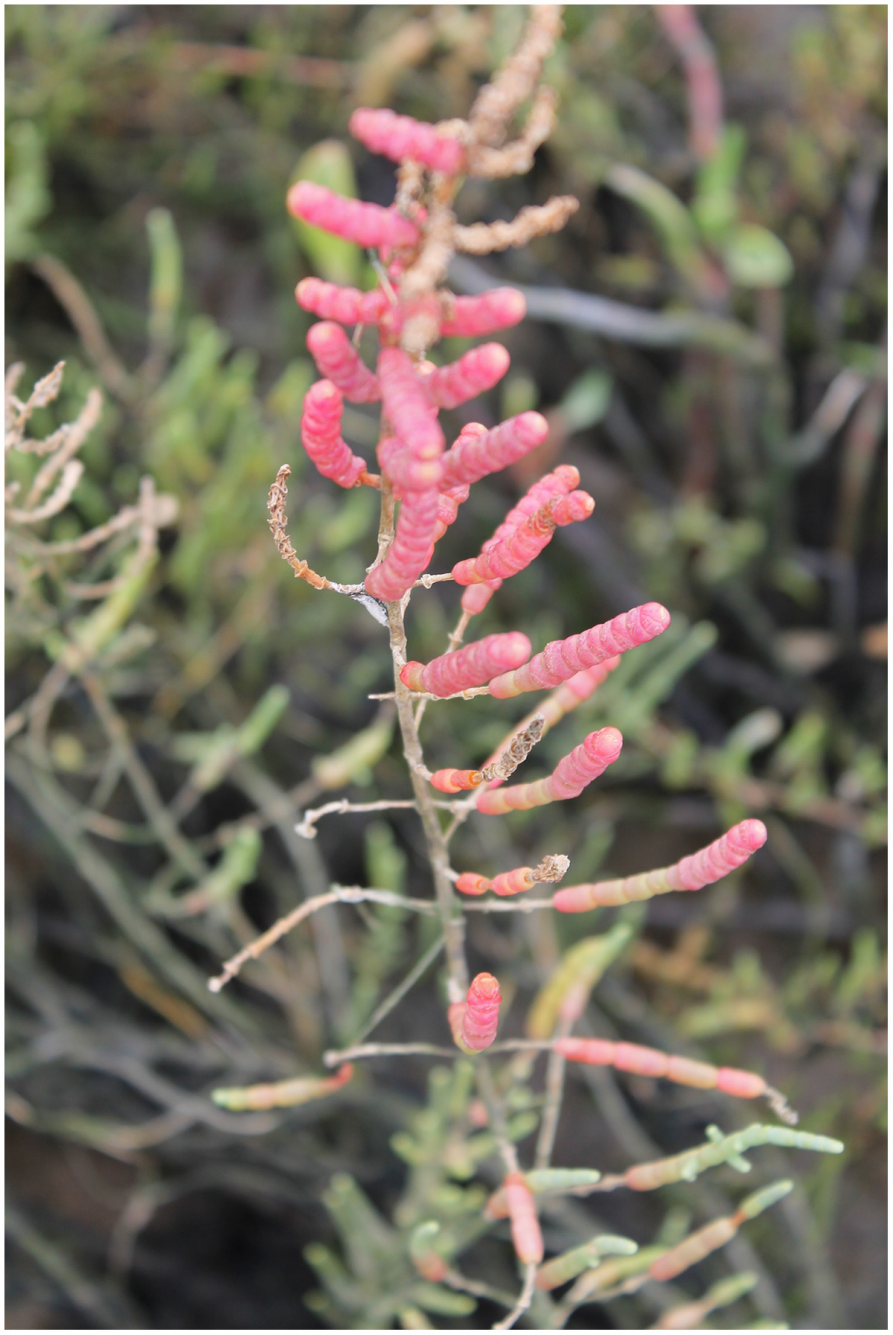
***Sarcocornia fruticosa* exhibiting a red coloration during the summer due to excessive salt concentrations in the sediments Photo by B. Duarte (2012)**.

**FIGURE 13:**
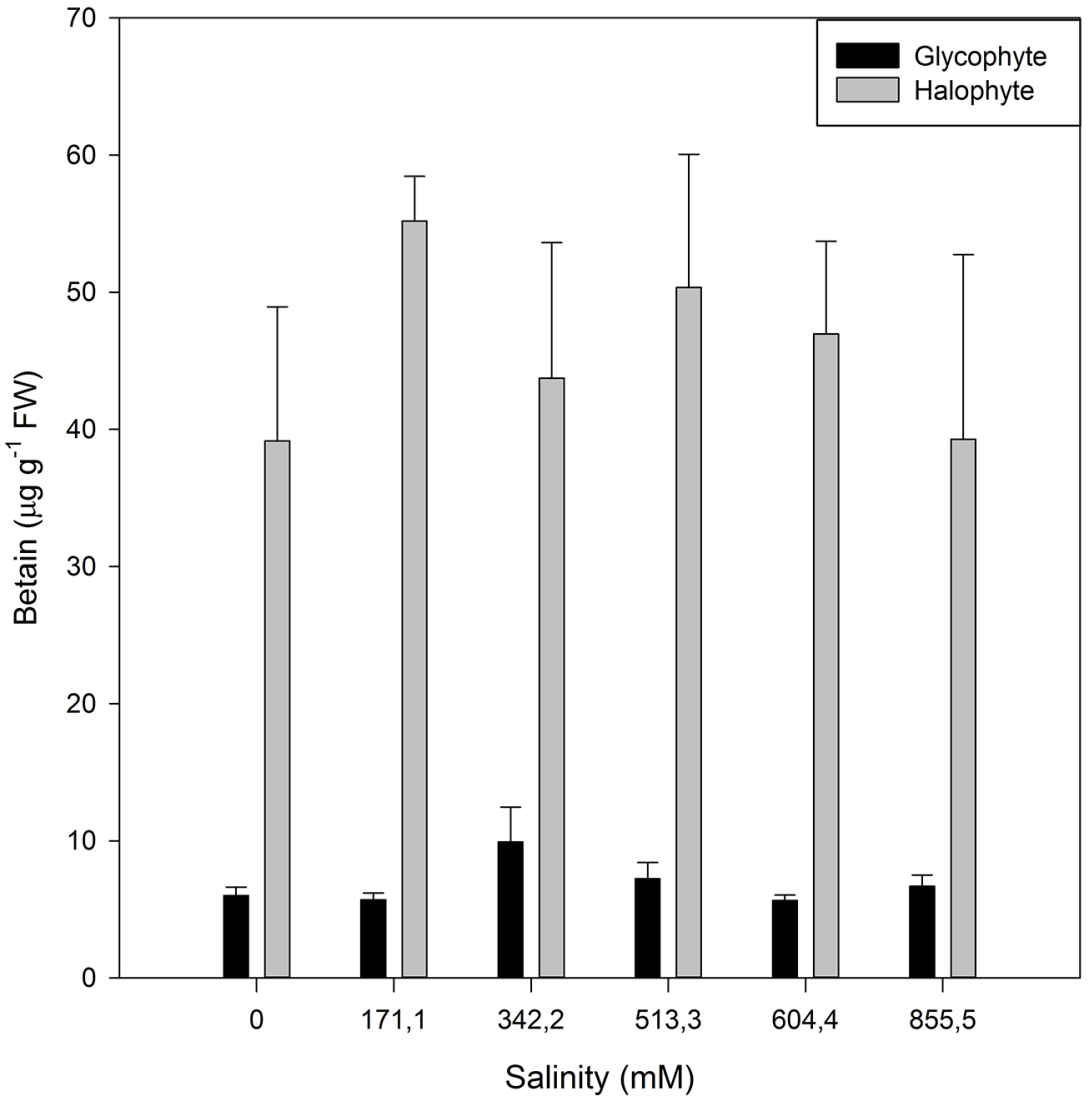
**Betain concentration in the leaves of a glycophyte (*Cyperus longus*) and of a halophyte (*Spartina patens*) exposed to a salinity gradient during 1 week (average ± SE, *N* = 5)**.

**FIGURE 14:**
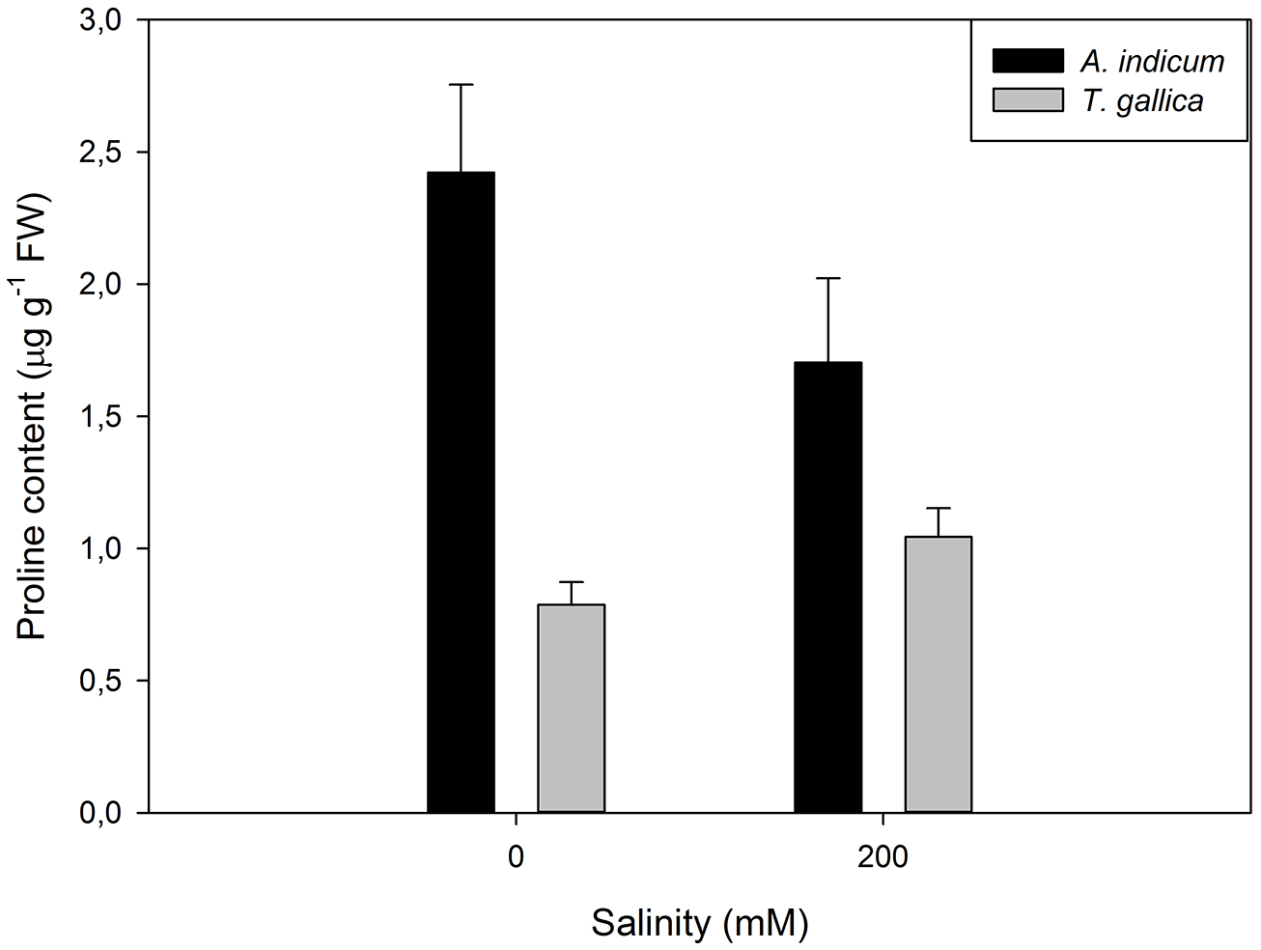
**Proline content in aboveground organs of *Arthrocnemum indicum* and *T. gallica* exposed to a salinity gradient during 20 days (average ± SE, *N* = 5)**.

Although this shift toward the carotenoid production is not evident by the naked eye, sometimes another phenomenon can be observed in large halophytic extensions, especially during summer. During warm seasons, sediment water evaporates increasing greatly the sediment salinity, to values sometimes twice the observed in seawater. Under these conditions, *Amaranthaceae* salt marshes frequently exhibit large areas of red-colored plants (**Figure 12**). This coloration is due to the presence of water-soluble pigments from the betacyanin family, normally produced as response to salinity, anoxia, or thermal stresses (Chang-Quan et al., 2006). Betacyanins play an important role in scavenging ROS, generated under environmental stress conditions (Stintzing and Carle, 2004). Chang-Quan et al. (2006) found similar results for other *Amaranthaceae* species (*Suaeda salsa*), suggesting that this betacyanin production is part of a common defense mechanism against environmental stresses, namely salinity. Commonly, these pigments are also related to a high betain production, a quaternary ammonium compound, mainly accumulated in the chloroplast in order to counteract high Na^+^ concentrations in this compartment (Rhodes and Hanson, 1993; McNeil et al., 1999). Again, comparing glycophytes (e.g., *Cyperus longus*) with halophytes (e.g., *Spartina patens*), the differences are evident (**Figure 13**). Halophytes are highly adapted to salinity, with an enormous production of betain in order to balance and regulate the osmotic potential inside its photosynthetic compartments. In glycophytes, these pathways are not well developed and thus the osmoregulation mechanisms are only adapted to small salinity fluctuations within an extremely low salinity range. Regarding the cytosol, the plant tends to accumulate proline, an amino acid with also a quaternary ammonium-based structure. In this cellular compartment, proline acts as an effective osmoregulator of the ionic pressure exerted by excessive salt concentrations. The use of this compatible solute can also reflect the salt tolerance strategy of a species. Comparing, e.g., an obligatory halophyte (*Arthrocnemum indicum*) with a salt-excreting facultative one (*T. gallica*) the differences are evident (**Figure 14**). While for *A. indicum* the absence of salt is an osmotic stress factor, in *T. gallica* the presence of salt, even at reduced concentration triggers the cytosolic accumulation of proline to counteract the osmotic imbalance. Allied with this compatible solute accumulation, *T. gallica* excretes the excessive salt from its leaves. In this case, the function of proline accumulation has a counteractive measure against the external medium osmotic pressure.

**FIGURE 15:**
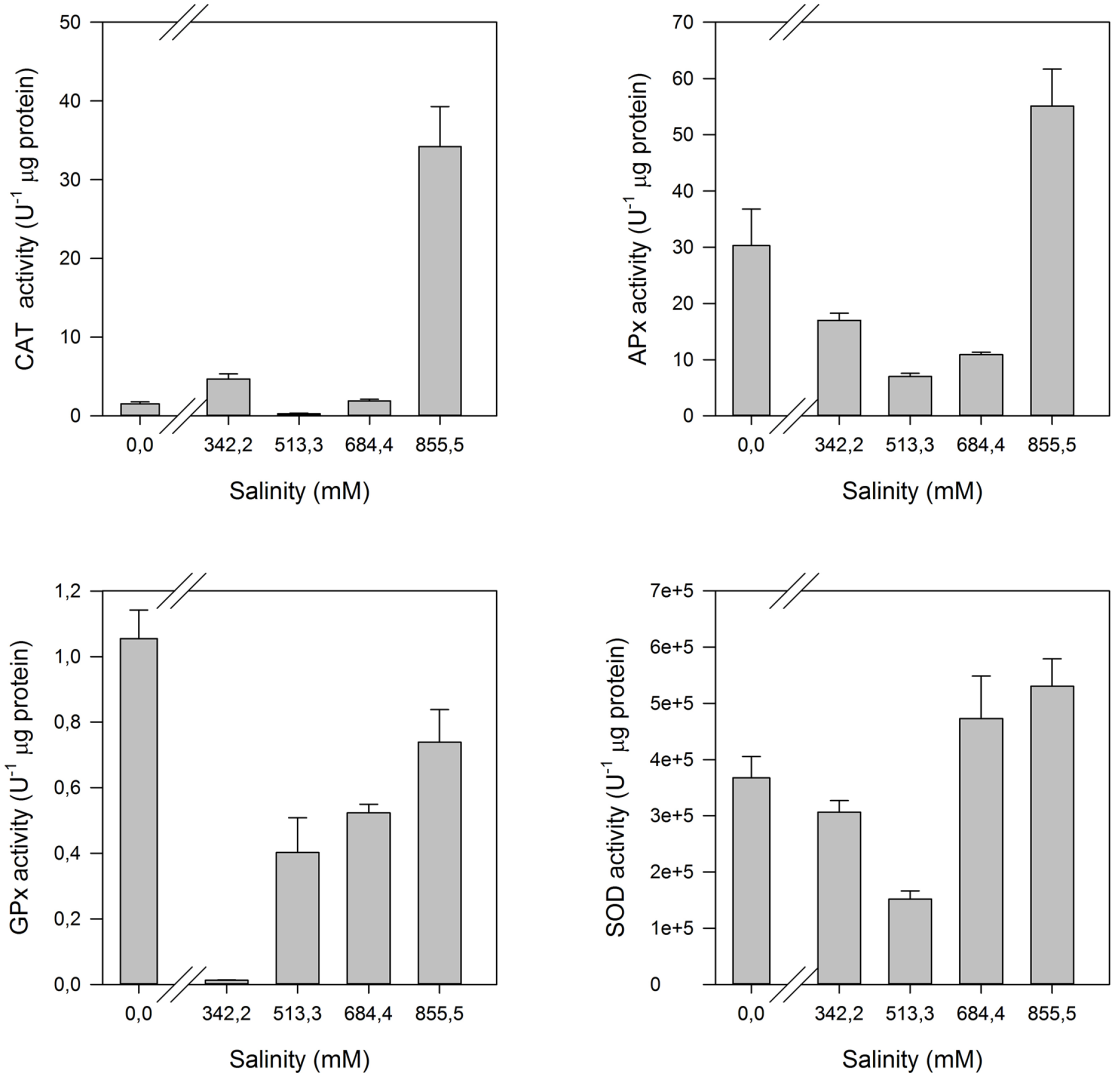
**Anti-oxidant enzymatic activities (CAT, Catalase; APx, Ascorbate Peroxidase; GPx, Guaiacol Peroxidase; SOD, Superoxide Dismutase) in the leaves of *H. portulacoides* exposed to a salinity gradient during 1 week (average ± SE, *N* = 5)**.

Halophytes are often classified as extremophile species, inhabiting extremely salinized and arid environments under extreme abiotic adverse conditions for life development. Another interesting adaptation developed by this group of plants was the acquisition and development of highly efficient battery of anti-oxidant enzymes. The interaction of high Na^+^ concentrations, as well as any other excessive cation concentrations, with the cell organelles lead to generated ROS resulting to reactions with proteins and the cellular biological compounds in membranes (Duarte et al., 2013c). Halophytes developed a highly efficient enzymatic rapid response system toward salinity changes, quickly activated when the medium conditions shift aside from the saline comfort zone of a halophyte (**Figure 15**).

This battery has its higher expression at the first line of defense, superoxide dismutase (SOD). This enzyme catalyzes the conversion of the highly toxic superoxide anions to hydrogen peroxide. In the second line of defense, peroxidase-class enzymes, such as catalase (CAT), ascorbate peroxidase (APx), and guaiacol peroxidase (GPx) play key functions in the hydrogen peroxide detoxification, and thus in the reduction of ROS to non-damaging concentrations. While for glycophytes it would be expectable that these defense mechanisms are activated with the increasing salinity doses, in halophytes the lack of salt can also be a stress factor, especially if we are dealing with obligate halophytes. Some authors suggest that obligate halophytes not only exhibit optimum growing in salt mediums, but in fact they require salt as part of their nutrition in order to activate or de-activate several salt sensitive enzymes (Wang et al., 2011). These species frequently exhibit an activation of these enzymes at both very low Na^+^ concentrations (below the physiological optimum) and at seawater Na^+^ concentrations (considered excessive), pointing out to a physiological Na^+^ dependence of certain halophytes, such as *H. portulacoides* (**Figure 13**).

## LEARNING FROM HALOPHYTES: FINAL REMARKS

Halophytes are extremely plastic species with a high degree of adaptation to saline habitats, being therefore excellent models to study salt resistance and tolerance mechanisms. Alongside, some halophytes have recently been pointed out as potential alternative cash crops for replacing usual crops in soils with excessive salt concentrations. Their tolerance to salt goes from simple morphological adjustments, like increasing turgescence or specific salt glands, to efficient energy dissipation mechanisms based on electron fluxes adjustment inside the chloroplast or to the production of specific molecules with the main objective to counteract the osmotic unbalance driven by excessive salt. Nowadays, the metabolic biophysical and biochemical mechanisms underlying these processes are relatively well described for several halophytes. This opens a new door where physiology can be allied to biotechnology, identifying the key genes underlying these processes and introducing them into non-tolerant crops. This will allow glycophytic species to be cultured in arid and saline lands maintaining the food supply in some of the poorest regions of the planet.

## Conflict of Interest Statement

The authors declare that the research was conducted in the absence of any commercial or financial relationships that could be construed as a potential conflict of interest.
